# Robustness of elastic properties in polymer nanocomposite films examined over the full volume fraction range

**DOI:** 10.1038/s41598-018-35335-1

**Published:** 2018-11-19

**Authors:** E. Alonso-Redondo, L. Belliard, K. Rolle, B. Graczykowski, W. Tremel, B. Djafari-Rouhani, G. Fytas

**Affiliations:** 1 Max Planck Institute for Polymer Research, Ackermannweg 10, 55128 Mainz, Germany; 2 Institut des NanoSciences de Paris, Sorbonne Universités, UPMC Universités Paris 06, UMR 7588, Paris, F-75005 France; 3 Johannes Gutenberg University, Institute for Anorganic and Analytical Chemistry, Duesbergweg 10-14, 55099 Mainz, Germany; 4 Institut d’Electronique, de Microélectronique et de Nanotechnologie (IEMN), UMR-CNRS 8520,UFR de Physique, Université de Lille 1, 59655 Villeneuve d’Ascq, France

## Abstract

Polymers with nanoparticle inclusions are attractive materials because physical properties can be tuned by varying size and volume fraction range. However, elastic behavior can degrade at higher inclusion fractions when particle-particle contacts become important, and sophisticated measurement techniques are required to study this crossover. Here, we report on the mechanical properties of materials with BaTiO_3_ nanoparticles (diameters < 10 nm) in a polymer (poly(methyl methacrylate)) matrix, deposited as films in different thickness ranges. Two well-known techniques, time and frequency domain Brillouin light scattering, were employed to probe the composition dependence of their elastic modulus. The time domain experiment revealed the biphasic state of the system at the highest particle volume fraction, whereas frequency domain Brillouin scattering provided comprehensive information on ancillary variables such as refractive index and directionality. Both techniques prove complementary, and can in particular be used to probe the susceptibility of elastic properties in polymer nanocomposites to aging.

## Introduction

Polymer nanocomposites are of current interest due to the possibility of tuning material properties by varying size and volume fractions of included nanoparticles^1,2^. In particular, the elastic modulus is an important property for applications and knowledge of the component moduli is not sufficient for predicting it, effective medium theories being often unreliable^3,4^. Thus, the combination of the two materials can sometimes exceed the bulk behavior of the constituents, owing to a synergistic interplay between the two components. More frequently however, unwanted side effects appear, because thermodynamically, polymer nanocomposites present a two component system which in the absence of specific interactions is virtually immiscible. To avoid the resulting non-equilibrium morphologies and obtain stable dispersions, a number of alternative strategies, e.g. polymer tethered particles^5–7^, exist. However, this and similar approaches require non-trivial chemistry as opposed to homogeneous hybrid materials fabricated by creating strong attachment of the nanoparticles to a polymer network^8^.

Where elastic behavior is concerned, if and how particles form contacts is known to be a determining factor, and fast acoustic techniques have recently been deployed to investigate the problem^9,10^. While giving insight into the transition between different chemical bonding regimes (van der Waals, covalent), a simpler and often more practical distinction is that between absence and presence of bonds altogether, depending on inter-particle distance. Studying this change in composition not only requires looking at a wider range of polymer fractions than previously considered, but can also benefit from a diversification of optical spectroscopy techniques. Time domain acoustic Brillouin scattering (also known as picosecond pump-probe acoustics)^11,12^ and frequency domain Brillouin Light Scattering (BLS)^13^ are presently the two most commonly utilized techniques for mechanical characterization at GHz frequencies and on the microscale. While frequently competing to study similar systems, to the best of our knowledge, the two methods have never been used complementarily. Polymer nanocomposites, due to their wide tunability, may however be expected to benefit from a multi-pronged approach to elastic characterization.

From our previous study^3^, we know that nanoparticle films can display lower sound velocities than their bulk constituent material, depending on the contact stiffness between the nanoparticles^4,14^. This was unexpected behavior, since polymer nanocomposites are generally stiffer than their constituent polymers^15–17^. However, the effect depends on the density of the filler of macroscopic and micrometer-sized composite particles^18,19^. Since the emphasis of the previous work was on superlattice type geometries, here, we focus on samples of BaTiO_3_ nanoparticles (diameters < 10 nm), with and without polymethyl methacrylate (PMMA) matrix, which we will examine by the two experimental techniques mentioned above. One single acoustic mode was observed by a wave vector q dependent BLS experiment that indicates a morphologically homogeneous polymer nanocomposite^16^. The phase sound velocity, however, was much lower than anticipated from effective medium models. This behavior was confirmed by a pump-probe experiment with access to single (backscattering) q for a sample of symmetric composition. We found that polymer nanocomposites are robust with respect to sound velocity and phonon lifetime for high polymer contents, but exhibit instability and pronounced aging effects for the highest particle fraction. A combination of two techniques – BLS and picosecond ultrasonics – is best adapted to probing the intermediate volume fraction range, revealed to have a biphasic character by measurement on thin samples.

## Results

### Spincoated samples

**Figure 1:**
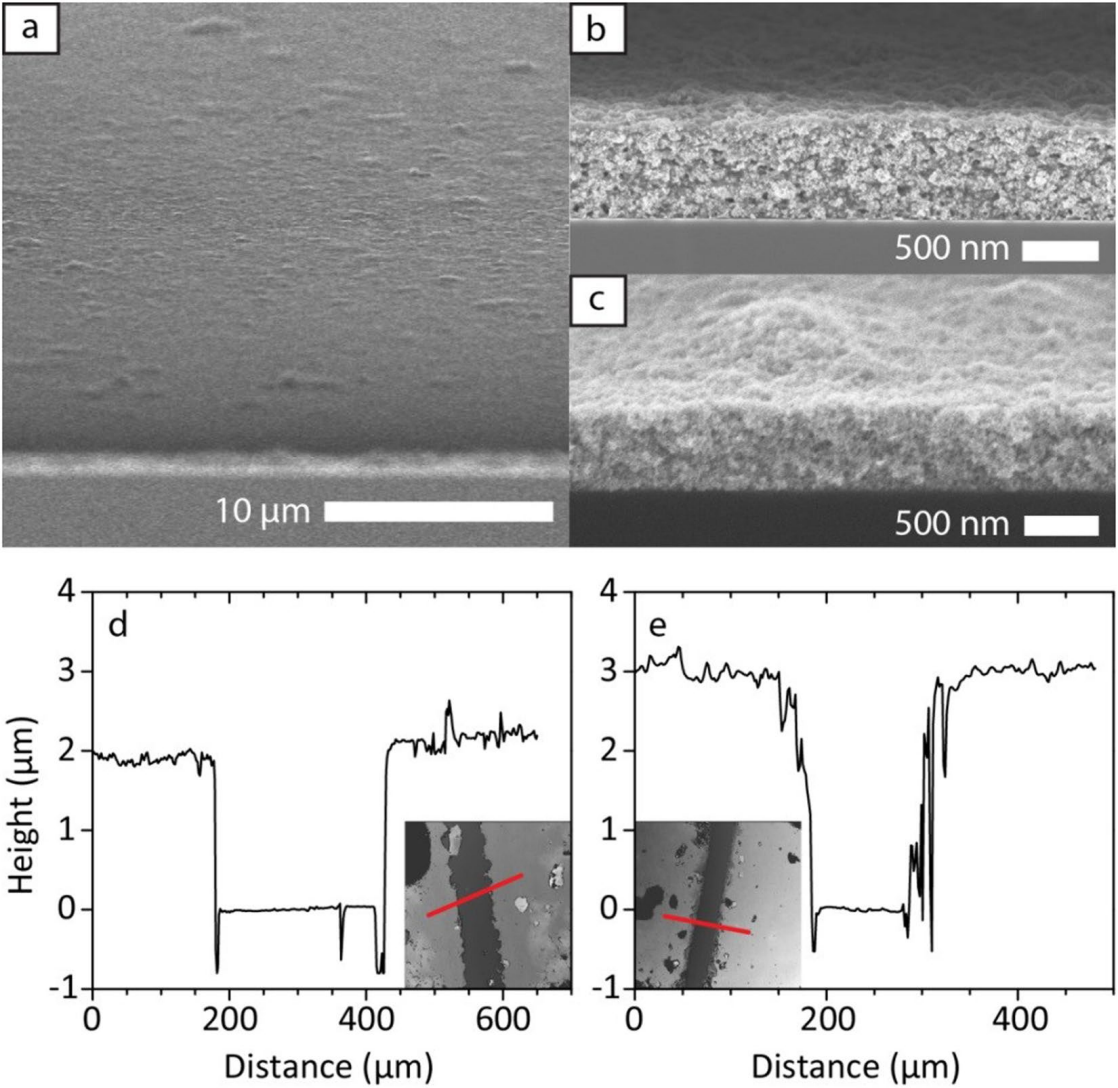
Polymer nanocomposite films. Cross-section SEM micrographs of (**a**,**b**) a PMMA-BaTiO_3_ and (**c**) a BaTiO_3_ film used in the picosecond ultrasonic experiment. The top surface of the PMMA-BaTiO_3_ film in (a) has a homogeneous thickness over the total area (circular spot of ≈20 µm diameter).

The films were prepared from PMMA and BaTiO_3_ nanoparticles solution by spin-coating. The BaTiO_3_ particles were synthesized hydrothermally^20^. Solutions with different concentrations of BaTiO_3_ and PMMA were spin-coated on substrates several times until the desired thickness was achieved. The films used in the BLS experiments were spin-coated on transparent glass substrates and annealed at 100 °C for 3 h (no vacuum), while the films for picosecond acoustics were spin-coated on silicon wafers. In the latter case, the lone PMMA-BaTiO_3_ film was prepared from equal parts (50 wt%) solution of BaTiO_3_ and PMMA. The exact thickness of the films was determined by scanning electron microscopy (SEM) shown in Fig. 1 and confocal microscopy (Fig. S1). The thicknesses for the picosecond acoustics films are found to fluctuate between 480–580 nm for pure BaTiO_3_, and 640–760 nm for PMMA-BaTiO_3_. For the BLS films, the average thickness was around 5 µm (Fig. S2).

### Brillouin light scattering (BLS)

**Figure 2:**
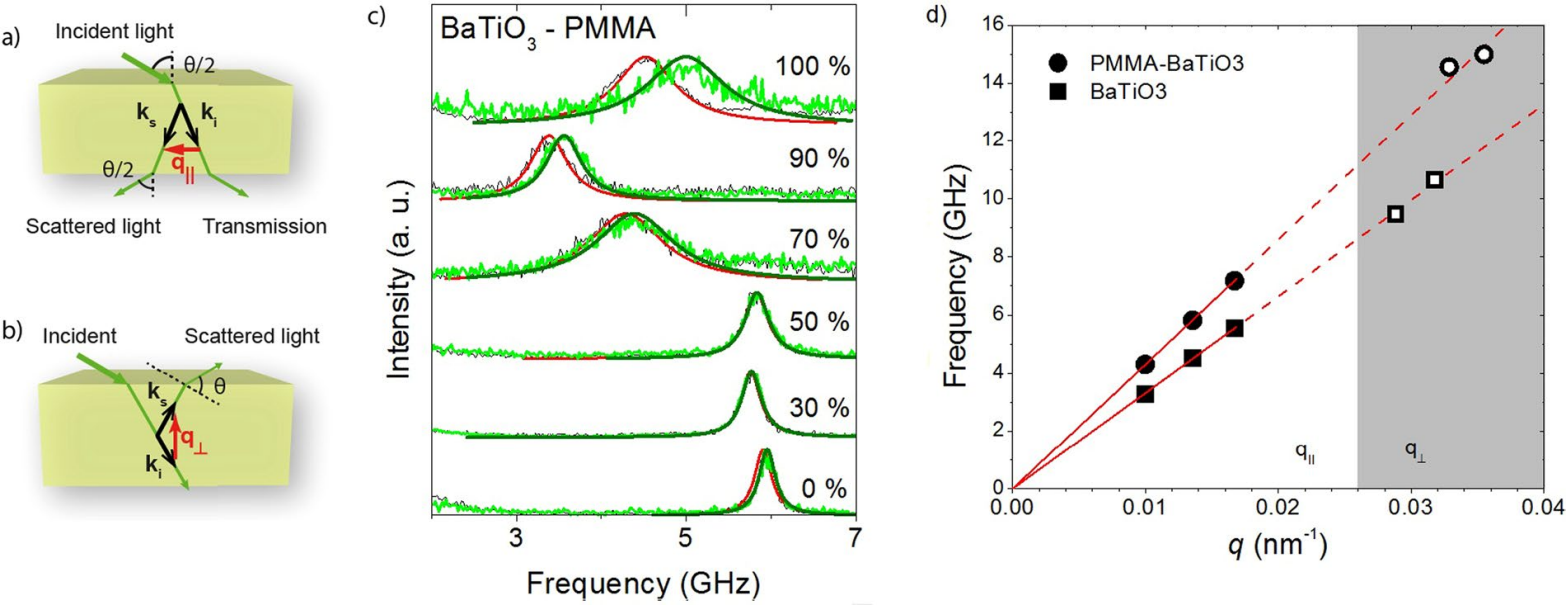
Brillouin light scattering experiment. (**a**,**b**) Scattering geometries in the BLS experiment, transmission (**a**) and reflection (**b**). (**c**) Polarized BLS spectra (anti-Stokes side) recorded in transmission geometry at q_*||*_ = 0.0135 nm^−1^ before (black) and after (light green) annealing. The peak has been fitted by Lorentzian curves, red and dark green, before and after annealing, respectively. (**d**) In-plane and out-of-plane (grey shaded area) phonon dispersion for BaTiO_3_ and PMMA-BaTiO_3_ (50 wt%). Filled (●, ■) and empty (○, □) symbols denote measurements on 5 and 2 [µm] thick samples respectively. See supporting information for spectra acquired using 2 [µm] thick samples.

In the (frequency domain) BLS experiment, the elasto-optic interaction of the incident light with thermally activated phonons causes a Brillouin shift in the light scattering spectrum. This frequency shift *f* is recorded as a function of the wave vector **q** to reveal the dispersion relation. The magnitude of the probing wave vector **q** depends on the scattering geometry. In transmission geometry (Fig. 2a), **q** lies in the substrate plane and its magnitude is *q*_‖_ = 4*π*/*λ*_*B*_sin*θ*_*B*_/2 where λ_B_ = 532 nm and θ_B_ is the scattering angle. In reflection geometry (Fig. 2b), **q** is perpendicular to the substrate plane, and its magnitude ${q}_{\perp }=4\pi /{\lambda }_{B}\sqrt{{n}^{2}-{\cos }^{2}{\theta }_{B}/2}$is dependent on the refractive index *n* of the sample^21^. The polarized BLS spectra at single wave number *q* acquired using transmission geometry are displayed in Fig. 2b for six films of PMMA-BaTiO_3_ composite and pure BaTiO_3_. Spectra are given for samples freshly drop-casted and annealed at 100 °C for 3 h under vacuum. The spectra were each fitted to a single Lorentzian curve in order to determine the peak positions and linewidths. The peak position as a function of *q* reveals the dispersion relation of the acoustic waves with phase velocity, *c* = 2*πf/q*. For clarity and easier comparison with the picosecond case considered below, dispersion relationships are given in Fig. 2d for two samples (50 wt% and 100 wt% BaTiO_3_) only.

Given that the dispersion is linear as observed for all wavenumbers probed in transmission geometry, the refractive index of the sample can be determined by recording the BLS spectrum in reflection geometry. Since the refractive index is needed for use with the picosecond acoustics experiment below, we thus determined it for two samples (50 wt% and 100 wt% BaTiO_3_), discussed in the supplementary information (Fig. S1). Refractive index values were found to be *n* = 1.56 (50 wt%) and *n* = 1.41 (100 wt%) at 532 nm, not far from our previous value for PMMA-BaTiO_3_ superlattices^3^.

### Picosecond acoustics

**Figure 3:**
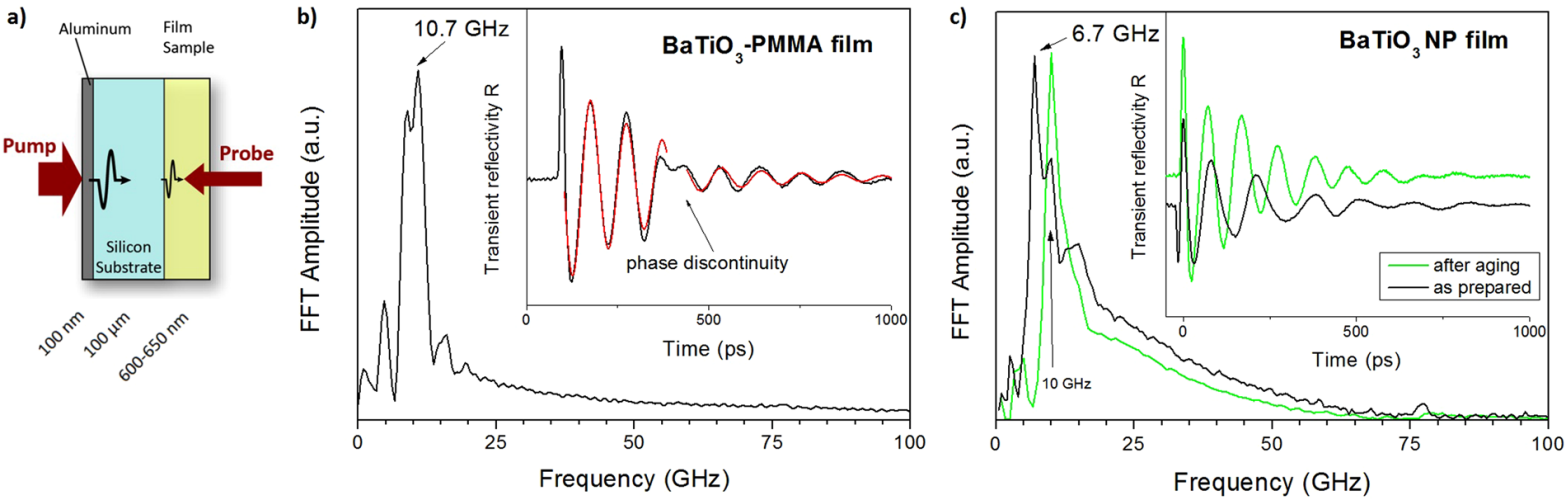
Picosecond pump-probe experiment. (**a**) Experimental schematic. (**b**,**c**) Inset: transient reflectivity as a function of the delay time for the (**b**) PMMA-BaTiO_3_ film and (**c**) two BaTiO_3_ films as prepared and after annealing. The transient reflectivity in (**b**) is represented by a damped sine shape (red line) and the arrow marks the change of phase due to the pulse arriving at the free surface of the film. Main**:** The fast Fourier transformation (FFT) of the transient reflectivity patterns in the insets to (**b**,**c**).

In this optical pump-and-probe method, an ultra-short pump laser pulse creates a sudden and small temperature rise. The induced thermal stress relaxes by launching a longitudinal acoustic strain field into the system^22^. The acoustic strain field is detected through the change of the sample reflectivity by a variable time-delayed laser pulse, commonly called the probe beam. In our experimental setup, the real and imaginary parts of the relative change of the sample reflectivity induced by the strain field can be measured by interferometric measurements^12,23,24^. To prevent sample damage induced by the pump energy, the acoustic transduction was carried out on a 100 nm thin aluminum layer evaporated on the backside of a silicon wafer. The thermal diffusion length is much lower than the thickness of the wafer, therefore heating induced by the pump can be neglected. After around 12 ns, the longitudinal pulse emerges at the silicon-film interface where the detection is performed across the transparent nanocomposite film (Fig. 3a). For the probe laser, a very low energy of 30 μW was utilized, again to preclude sample deterioration. Indeed, no signal fluctuation was observed during the averaging process (though for the volume fractions dominated by PMMA, which is in the glassy state, the effect of heating on sound velocity would anyhow be expected^25^ to be very weak, around dc/dT ~5 × 10^−4^ K^−1^). The beam is scanned over the sample surface in order to locate the pump epicenter, where a time-resolved measurement is done. The detection is performed perpendicularly to the film and at room temperature.

Figure 3b,c present the real part of the reflectivity after the echo emergence in the film sample. Following the sharp echo signature, the acoustic feature is characterized by typical oscillations, so-called Brillouin oscillations, resulting from interferences between light reflected by the sample-wafer interface and by the acoustic pulse propagating into the nanocomposite film^26^. The number of Brillouin oscillations of the signal before the jump in phase, due to the reflection of the acoustic pulse at the free surface of the film, corresponds to the number of acoustic wavelengths in the Fabry-Perot cavity formed by the free surface and the transducer interface. This number amounts to 2*nd*/λ_P_ where *d* is the thickness of the film, *n* the refractive index and λ_P_ = 800 nm the probing wavelength. In the case of the PMMA-BaTiO_3_ film (Fig. 3b) the number of oscillations is ~2.5 and in the case of the BaTiO_3_ film (Fig. 3c) it is ~2.2, which agrees well with the observed time domain waveform.

The Brillouin frequency (*f*_*B*_) is obtained by representing the transient reflectivity data (inset to Fig. 3b,c) by the damped sinusoidal function ${e}^{-t/{\tau }_{0}}\,\sin (2\pi ft+a)$, where *a* is the phase and τ_0_ the decay time. The Brillouin frequency can be also obtained directly from the fast Fourier transformation (FFT) of the transient reflectivity as indicated by the arrows in Fig. 3b,c. The spectral resolution of the FFT is rather poor, due to the small number of Brillouin oscillations before the back reflection, with a broad peak and additional peaks of smaller amplitude. The Brillouin frequency amounts to 10.7 GHz and 6.7 GHz respectively for the as-prepared PMMA-BaTiO_3_ (50 wt%) and BaTiO_3_ film. Unexpectedly, aging influenced only the latter particle film as witnessed by the blue shift of its Brillouin frequency to be discussed in the next section. The subsequent calculation of the sound velocity from^27^ f_B_ = (2nc/λ_P_)sin(θ_P_/2), with θ_P_ being the angle between incident and reflected probe light, requires independent access to the refractive index n; backscattering θ_P_ = 180° was experimentally utilized. Assuming negligible dispersion of n(λ) in the range 532–800 nm, we use *n* obtained from BLS measurements on out-of-plane acoustic propagation to compute the longitudinal sound velocity in BaTiO_3_ and PMMA-BaTiO_3_ (50 wt%). A comparison of the sound velocity and the attenuation values from both techniques is given in Table 1, with linewidth values for BLS given after subtraction of the convoluted instrumental response (intercept in Fig. S4). For the pump-probe experiment, the decay times τ_0_ cannot be directly compared, because the wave vectors q = 0.0221 nm^−1^ and q = 0.0245 nm^−1^ (calculated from q = 4πn/λ_P_) are different due to different refractive indices of the samples. Since the half-width at half-height scales with q^2^ (cf. Fig. S4 for BLS), we listed the extrapolated value at q = 0.0135 nm^−1^ corresponding to the wave vector of the BLS experiment. For the polymer nanocomposite film with 50 wt% composition, the phonon mean free path (≈*cΓ*) obtained from the two techniques is quite similar. For the pure BaTiO_3_ nanoparticle case however, the phonon mean free path is much shorter (larger *Γ*) in the samples used in BLS than in the pump-probe experiment, suggesting significant phonon scattering in the BLS samples. It should be noted that the estimated phonon lifetime represents an upper bound as dephasing of the signal due to spatial inhomogeneities has been neglected. In addition, the phonon lifetime from the pump-probe experiment is bound to increased errors as the number of the Brillouin oscillations decreases.

**Table 1:** Comparison of acoustic parameters of similarly composed films measured with the pump probe and BLS experiments.

	Parameter	PMMA-BaTiO_3_	BaTiO_3_
Picosecond acoustics	*c* (m/s)	2740 ± 60	1900 ± 140 (2800 ± 200)
	*Γ* = 1/(2*πτ*_*0*_) (MHz)	550 ± 40	640 ± 30
	*Γ* (MHz) @ q = 0.0135 nm^−1^	167 ± 12	239 ± 11
BLS	*c* (m/s)	2700 ± 15	2090 ± 10
	*Γ* (MHz) @ q = 0.0135 nm^−1^	120 ± 8	503 ± 55

Parenthesized value from small side peak in Fig. 3c.

## Discussion

**Figure 4:**
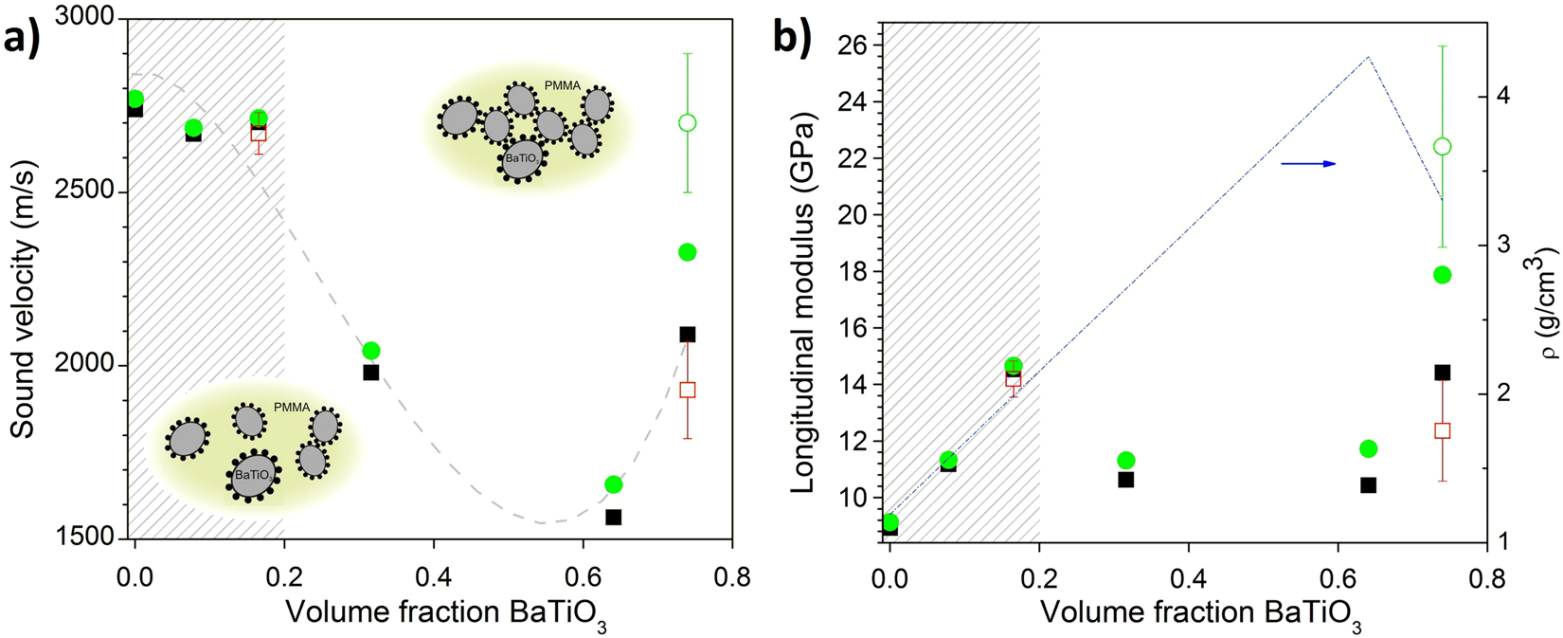
Volume fraction dependence. (**a**) Sound velocity against volume fraction for all 6 films^3^. Dashed line guide to the eye only. (**b**) Corresponding longitudinal modulus. Blue line shows density, with the deviation at high volume fraction due to porosity^3^. Square symbols (■, □) represent data points from “as prepared” and round ones (●, ○) from “annealed” or “aged” samples. Filled (●, ■) and empty (○, □) symbols denote data points from BLS and picosecond acoustics measurement respectively. The structures at low and very high volume fraction are schematically shown in the insets to (**a**).

Comprehensive investigation of composition-dependent behavior requires consistency between samples for comparison purposes, which makes thin films, afflicted by weak signals and large relative thickness fluctuations, a poor choice. Conversely, thicker films require many repetitive spin-coating steps rendering defect formation and hence inhomogeneity probable. Nevertheless, the BLS study over the full composition range was performed using about 5 µm thick films, and the variation of the sound velocity with nanoparticle volume fraction is depicted in Fig. 4. Corresponding to the BLS spectra from Fig. 2c, we can distinguish robustness of polymer nanocomposite properties for the three polymer-rich compositions with less than about 20 vol% hard component (shaded grey area). In this regime, the computed longitudinal modulus reveals the anticipated hardening. However above this filling ratio, lower sound velocities are observed in Fig. 2c, as well as increased sensitivity to annealing. Additionally, the spectra reveal a substantial linewidth broadening that seems to accompany this crossover behavior.

Turning to thinner films, we crosschecked the results for two compositions utilizing the picosecond acoustics technique. We note that BLS cannot be straightforwardly used for films thinner than 1 μm due to the spatial confinement effect and small scattering volume^3^. For the symmetric weight composition picosecond measurements are consistent with BLS results as is the case for bulk PMMA films (Fig. S3). For the as-prepared BaTiO_3_ film, however, the pump-probe experiment reveals a second peak at 10 GHz in addition to the main peak at 6.7 GHz (Fig. 3c). The existence of this peak, located at a frequency close to that of the main peak in the PMMA-BaTiO_3_ composite, is interpreted as evidence of biphasic regions in the sample. Its Brillouin frequency has been listed in Table 1 separately in parentheses. This double phonon structure is not discernible in the BLS spectrum of a thicker “as-prepared” BaTiO_3_ film that displays a broad but single spectral line (Fig. 2b, but note that laser spot size for the BLS measurement was also larger). The obtained sound velocity in the thicker “as-prepared” BLS measurement falls between the two values of the pump-probe experiment (Table 1, Fig. 4a). Interestingly, the structural heterogeneities in the “as-prepared” BaTiO_3_ film are absent in the polymer nanocomposite and might hint at a granular-like behavior, where contacts between BaTiO_3_ nanoparticles become important. Between 20–65 vol%, the sound velocity decreases with the solid fraction, whereas the modulus is virtually composition independent due mainly to the density increase.

BaTiO_3_ particles can interact via surface polycondensation in the binary mixture of PMMA and BaTiO_3_ particles due to reaction with water molecules in humid air to form surface hydroxyl groups^28^; the bridging OH groups are acidic, while the terminal OH groups are basic^29^. This leads to surface condensation (aging) and therefore particle aggregation, which is suppressed in polymer matrices, because there is no direct contact between particles. For sufficiently high volume fractions of the BaTiO_3_ particles, their effective attraction can cause microphase separation into dense BaTiO_3_ aggregates (“clustering”) and a dilute PMMA phase for compositional ratios between 20–65%. For low (<20%) volume fractions the interaction probability of the BaTiO_3_ particles is too low to lead to a significant effect. Above about 65%, the maximum packing density (of ≈64%) is reached for particles arranged at random positions. The distribution of microregions can be either random or build a gradient from the surface to the substrate. These extreme film morphologies can be discriminated from the present experiment with very few Brillouin frequencies (Fig. 3c)^30,31^.

The crossover behavior in Fig. 4 was verified by both naturally and artificially aging the samples, in picosecond acoustics and BLS respectively. In Fig. 3c the small side peak at 10 GHz in the ‘biphasic’ sample is thus seen to increase in amplitude upon aging, and annealing of the BLS samples is seen to affect only samples with higher BaTiO_3_ fractions. The absence of a significant response to aging proves that for low particle fractions, the polymer nanocomposite system is in quasi-equilibrium. For higher inclusion volumes, the change in behavior is in line with what can be expected if a transition from van-der-Waals to covalently bonded particles takes place, as studied elsewhere^9,10^. Finally, we see that robustness to aging effects is achievable by lowering nanoparticle fractions (see insets to Fig. 4a).

To make a prediction about sound velocity behavior as a function of volume fraction (Fig. 4), it would be necessary to make a detailed analysis of the morphology^32^ and then hypothesize about the way the elastic waves are propagating in the material. This is out of the scope of this paper, but we can address a few possibilities: If we assume first that spherical BaTiO_3_ nanoparticles are embedded in a PMMA matrix with a perfect elastic contact, the calculation of the effective sound velocity in a face centered cubic crystal reveals a very slight decrease of the sound velocity as a function of the filling fraction despite the high elastic impedance contrast (Δ(ρc_L_)) between BaTiO_3_ and PMMA^3^. This is in good agreement with the experimental trend at relatively low volume fraction but does not explain the significant drop of the velocity observed at higher volume fraction in Fig. 4. This corroborates the notion that the arrangement of the nanoparticles in the PMMA has been subject to some modifications. As already mentioned, the particles may attach to each other with small contact area and hence the transmission between them may involve other contact mechanisms than elastic transmission as well particle-particle interactions^33,34^. Additionally disorder in the NP chains, e.g. zigzag shapes and holes, can also affect the sound propagation. All these scattering mechanisms would be able to contribute to a drop of the sound velocity. Nevertheless, other scattering mechanisms may be active and compete as well. Therefore, an investigation of different models based on a detailed experimental analysis of the morphology, to discriminate each contribution, should be the object of a study by its own.

## Conclusion

Polymer nanocomposites show instability for high nanoparticle volume fractions, meaning decreased phonon lifetime and greater susceptibility to sample aging. Both BLS and picosecond acoustics can elucidate these phenomena, with the latter profiting from higher resolution afforded by its capability to measure samples that are thinner and by implication have weaker signals. BLS is better adapted to measurement of bulk samples, with only minimal preparation and no need for external phonon injection to boost signal. Thus, we were able to evidence the existence of biphasic regions using picosecond acoustics, and obtain a comprehensive picture covering different composition fractions using BLS. We hope these results will be useful to engineers having to tune polymer material properties for applications while assuring elastic robustness, and material scientists having to characterize new types of polymer nanocomposites.

## Methods

### Film preparation

Films were prepared from solution by the spin-coating technique (5000 rpm, 20 s, 5000 rpm/s). The BaTiO_3_ particles were prepared by a hydrothermal synthesis using barium (*Alfa-Aesar*) and titanium isopropoxide (*Acros*)^20^. The synthesized particles are approximately spherical and their size is 7 ± 1 nm, determined by transmission electron microscope (*JEOL 14*0*0*). The PMMA-BaTiO_3_ films were prepared from varying parts of a solution of BaTiO_3_ (3.4 wt% in ethanol) and solution of PMMA (35000 g/mol; 3.4 wt% in toluene), while for the BaTiO_3_ films a single solution of BaTiO_3_ nanoparticles was used. The solutions were spin-coated several times on glass (silicon) substrate in the case of BLS (picosecond acoustics) technique, until the desired thickness is achieved. For BLS, the thickness is about 5 µm in order to avoid out-of-plane elastic excitations and measure a single longitudinal acoustic mode. After each layer, the sample is dried at 80 °C for 20 min. The thickness of the films is determined by SEM (*Zeiss LEO Gemini*) and confocal (*Nanofocus*) microscopes.

### Brillouin light scattering

The probed samples are held in the center of a goniometer, to which the probing laser (532 nm wavelength) is attached, in order to record the spectra at several scattering angles. The polarized scattered light is collected by an arrangement of lenses and directed to the tandem Fabry-Perót interferometer (*JRS Instruments*). The Brillouin frequencies represented as a function of the probing q reveal the dispersion relation, which is linear in the case of the homogeneous films.

### Picosecond acoustics

The experimental setup is based on a mode-locked Ti:Sapphire (*MAI-TAI Spectra*) laser source operating at 800 nm, with a repetition rate of 79.8 MHz. The pulse time width is less than 100 fs at the laser output. The pump beam is modulated at 1.8 MHz to perform synchronous detection on the sample reflectivity. Using a detection scheme based on a Michelson interferometer, the real and the imaginary part of the transient reflectivity change is recorded. The fast Fourier transformation (FFT) of the transient reflectivity (insets to Fig. 3b,c) reveals the spectral information shown in Fig. 3b,c. Due to the small thickness of the sample, the number of Brillouin oscillations is small before the back reflection, and consequently the spectral resolution of the fast Fourier transform (FFT) is poor, with a broad peak and additional peaks of smaller amplitude. Additionally, by fitting the damped sinusoid piecewise (see Fig. 3b), we notice that the Brillouin frequency after the jump in phase is lower by 0.5 GHz (BaTiO_3_) and 1.2 GHz (PMMA-BaTiO_3_). This may be explained as follows: Due to the small thickness of the system, we only observe very broad Brillouin signatures. The rough sample surface (Fig. 1) may induce a lower specular reflection for the high frequency components of this broad signature, leading to harmonic distortion in the time domain waveform. The fit, which assumes a pure sine wave, then interprets this distortion as a frequency reduction. Thus, roughness influence on the specular reflection of acoustic pulses in GHz domain has already been reported^35^. This is probably also the reason why we do not observe standing waves as in some similar nanocomposite systems^10,14,36^. A jump of the transient reflectivity in the case of PMMA-BaTiO_3_ (arrow in Fig. 3b) located at 316 ps could be used to obtain the sound velocity given that the film thickness is well known^37,38^. This jump is related to modifications induced by the strain pulse in the probe optical path. Unfortunately the polymer layer exhibits thickness fluctuation (≈130 nm) which makes this determination tricky.

## Electronic supplementary material

Supplementary information
