# Supplementary material for: Robustness of elastic properties in polymer nanocomposite films examined over the full volume fraction range
Source: Sci Rep. 2018 Nov 19;8:16986. doi: 10.1038/s41598-018-35335-1 (PMC6242885; doi:10.1038/s41598-018-35335-1)
Supplement: Supplementary file 1 — Supplementary information [file 41598_2018_35335_MOESM1_ESM.docx]

# Supplementary information for “Robustness of elastic properties in polymer nanocomposite films examined over the full volume fraction range”

E. Alonso-Redondo, L. Belliard, K. Rolle, B. Graczykowski, W. Tremel, B. Djafari-Rouhani and G. Fytas


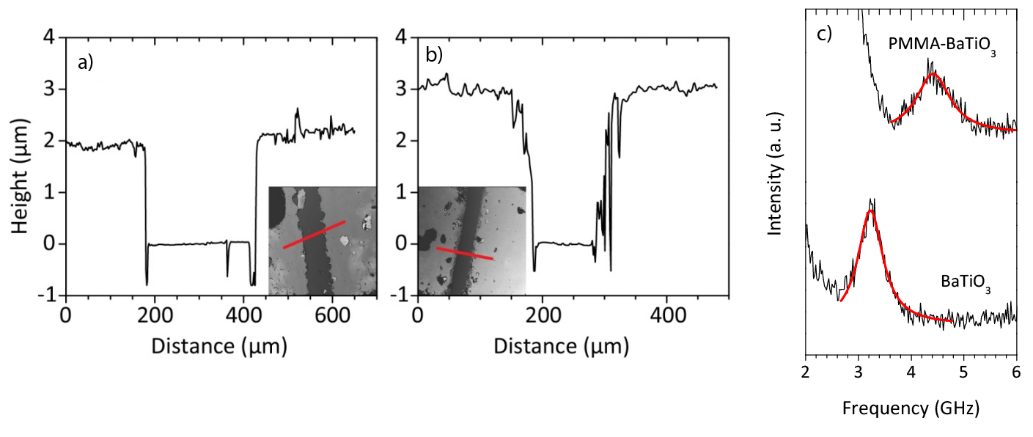


**Figure S1. Samples and spectra for 2 μm thick samples.** (**a-b**) Confocal profile of a scratch in the films used in BLS experiments reveals the thickness of (**a**) PMMA-BaTiO_3_ film, and (**b**) BaTiO_3_ film The insets display the microscopic image of lateral size 800 µm and the line of the profile in red. (**c**) BLS spectra at q*_||_* = 0.010 nm^-1^, showing broad peaks when compared to Fig. 2c (out-of-plane spectra used for refractive index determination not shown).

**a)**
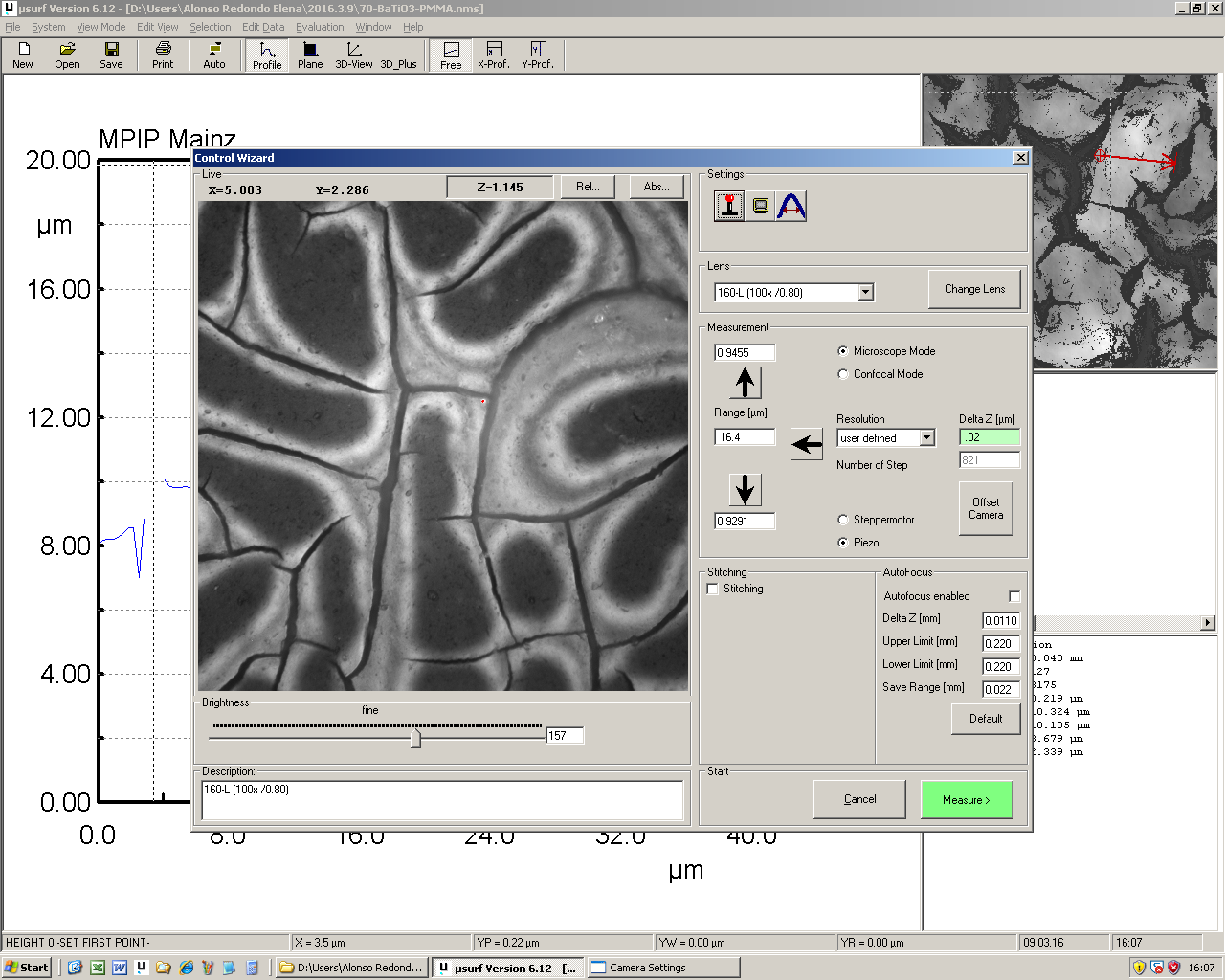
 **b)**


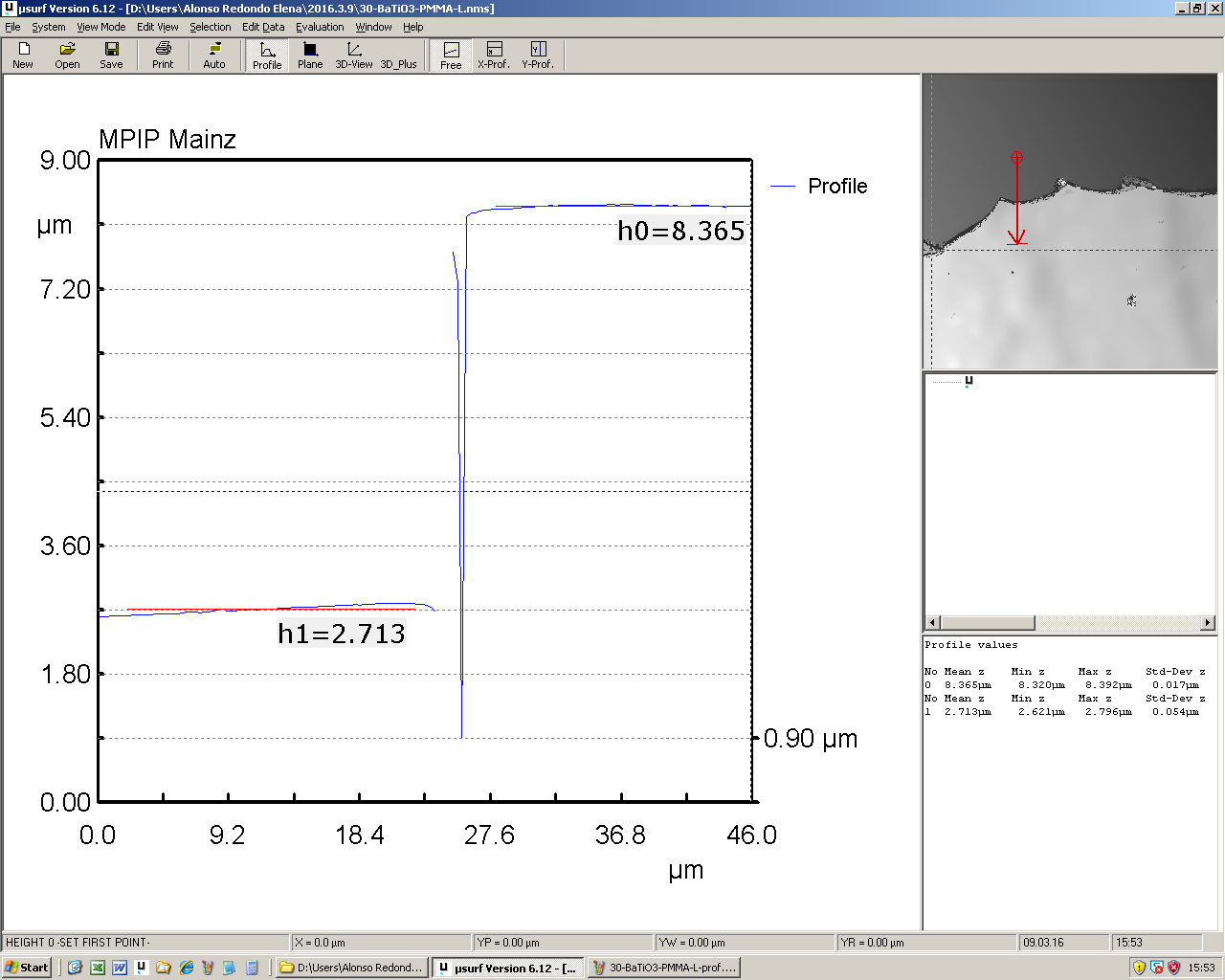

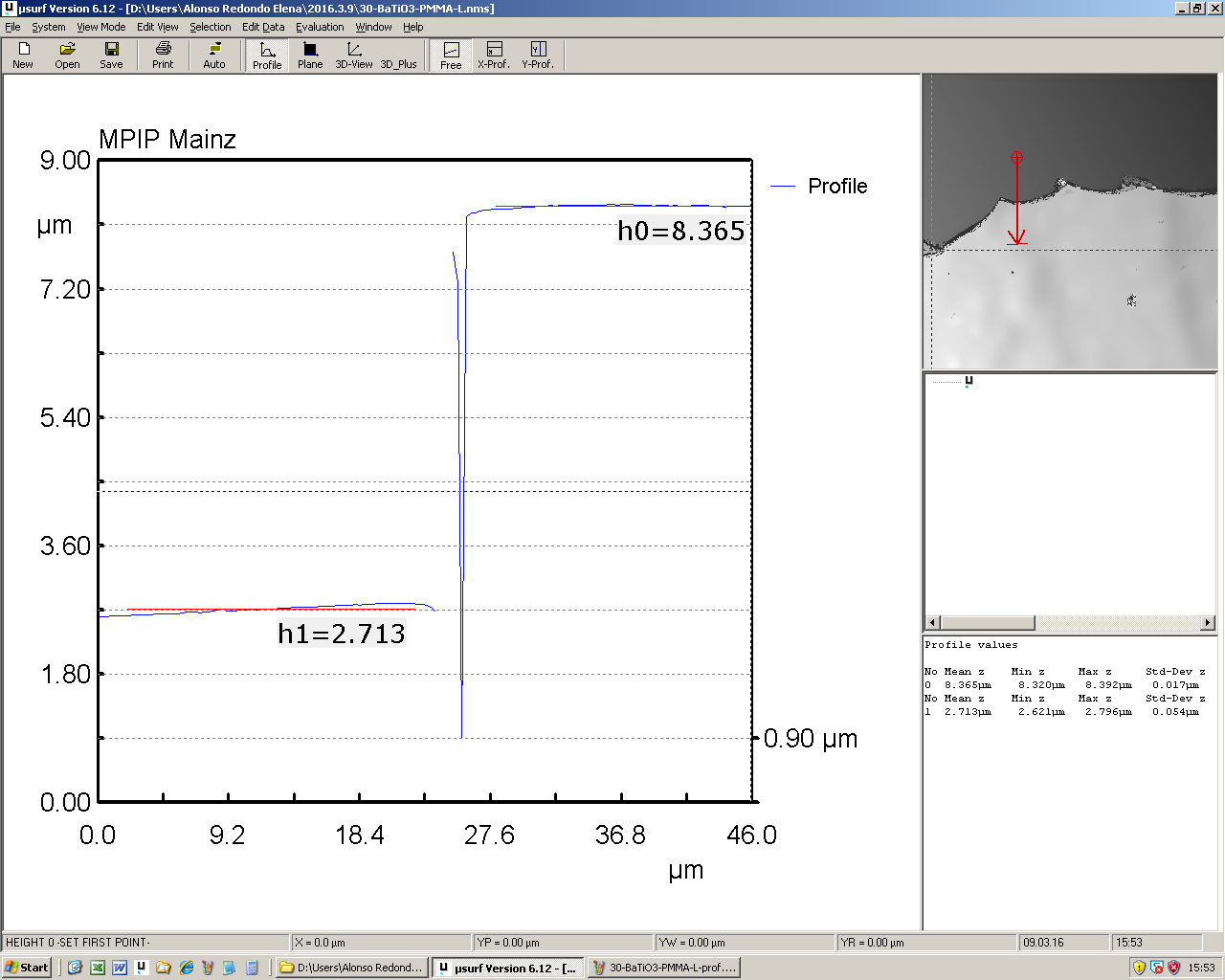


**Figure S2. Samples used for BLS study on volume fraction dependence.** (**a**) Microscope top view of the 90-10 BaTiO_3_-PMMA. (**b**) Height profile of the 30-70 BaTiO_3_-PMMA. The average thickness of all samples is about 5 µm.


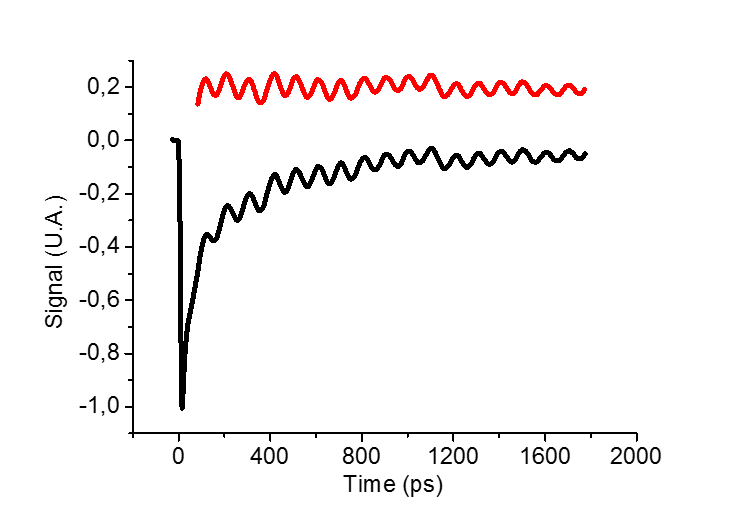


**Figure S3. Picosecond acoustic measurement of pure PMMA.** Black: PMMA layer reflectivity in reflection geometry. Red: Brillouin oscillation (9.8 GHz) after thermal background subtraction.


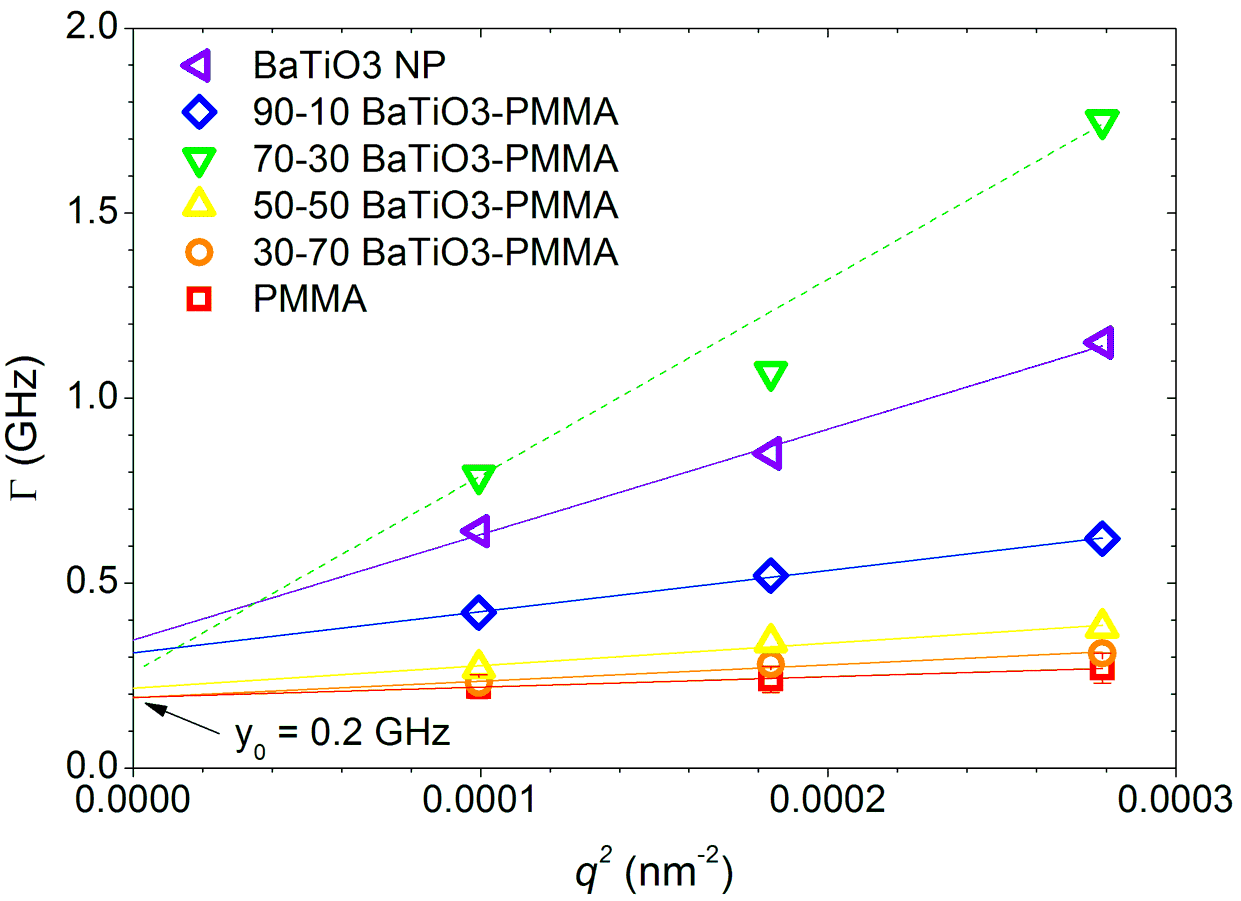


**Figure S4.** **FWHM from BLS spectra against q^2^**


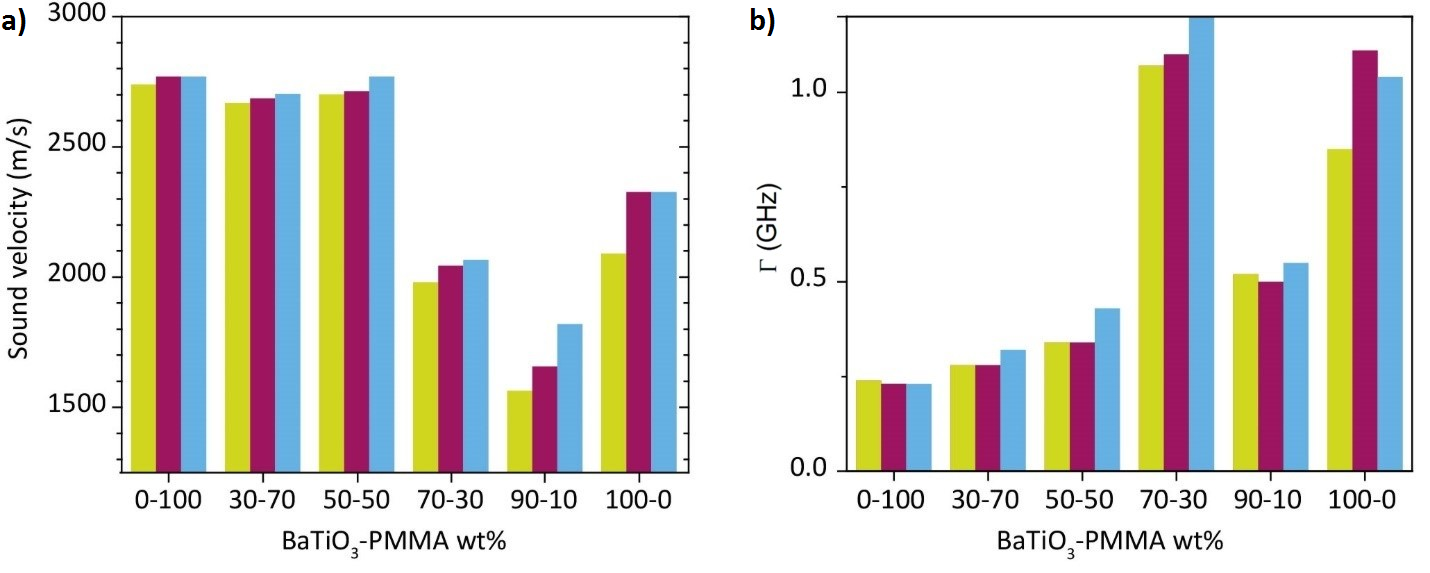


**Figure S5.** Evolution of the (**a**) sound velocity and (**b**) linewidth after annealing. Lemon = as received, purple = after annealing below T_g_, blue = after annealing above T_g_.
